# Treatments for Severe Cutaneous Adverse Reactions

**DOI:** 10.1155/2017/1503709

**Published:** 2017-12-27

**Authors:** Yung-Tsu Cho, Chia-Yu Chu

**Affiliations:** Department of Dermatology, National Taiwan University Hospital and National Taiwan University College of Medicine, Taipei, Taiwan

## Abstract

Severe cutaneous adverse reaction (SCAR) is life-threatening. It consists of Stevens-Johnson syndrome/toxic epidermal necrolysis (SJS/TEN), drug reaction with eosinophilia and systemic symptoms (DRESS), acute generalized exanthematous pustulosis (AGEP), and generalized bullous fixed drug eruptions (GBFDE). In the past years, emerging studies have provided better understandings regarding the pathogenesis of these diseases. These diseases have unique presentations and distinct pathomechanisms. Therefore, theoretically, the options of treatments might be different among various SCARs. However, due to the rarity of these diseases, sufficient evidence is still lacking to support the best choice of treatment for patients with SCAR. Herein, we will provide a concise review with an emphasis on the characteristics and treatments of each SCAR. It may serve as a guidance based on the current best of knowledge and may shed light on the directions for further investigations.

## 1. Introduction

Drug hypersensitivity may result in several different kinds of reactions. In most of the cases, drug hypersensitivity presents as generalized maculopapular exanthema, which is mild and almost self-limited after withdrawing the causative agents. However, in a small fraction of the cases, drug hypersensitivity would show up as a severe drug reaction. These severe reactions are life-threatening and termed as severe cutaneous adverse reactions (SCARs).

SCARs consist of some different disease entities, including Stevens-Johnson syndrome/toxic epidermal necrolysis (SJS/TEN), drug reaction with eosinophilia and systemic symptoms (DRESS), acute generalized exanthematous pustulosis (AGEP), and generalized bullous fixed drug eruptions (GBFDE) [1]. All of them harbor considerable rates of morbidities and mortalities. However, each SCAR has its own characteristic cutaneous presentations, causative drugs, clinical courses, pathomechanisms, and possible treatment modalities. Therefore, being familiar with SCARs and providing prompt treatments are important to manage these diseases and to reduce the adverse impacts. For this purpose, in this review, we will summarize concise descriptions regarding the characteristics of each SCAR with an emphasis on the options of treatment for each SCAR.

## 2. Stevens-Johnson Syndrome (SJS) and Toxic Epidermal Necrolysis (TEN)

### 2.1. Basic Characteristics

SJS and TEN are among the most important and well-known SCARs. The incidence of SJS/TEN has been reported to be 1.5–1.8/per million persons per year [2]. They are usually caused by a limited number of drugs, including anticonvulsants, sulfa-containing drugs, antibiotics, nonsteroidal anti-inflammatory drugs, and uric acid-lowering agents [3]. Patients with SJS/TEN usually develop mucosal erosions or ulcers with variable extents of skin detachment after ingesting causative agents for a period of 1–3 weeks [4]. The mucosal lesions may include the oral cavity, lips, conjunctivae, and genital areas. Skin lesions are usually widespread with a predilection on the trunk and consist of atypical flat target lesions, which may become confluent or result in the formation of blisters [5]. Systemic symptoms may develop, which include fever, general malaise, flu-like symptoms, and possible internal organ involvement [6].

SJS and TEN are thought be a spectrum of the same disease. They are classified, by the definition, using the extent of blistering or detachment in relation to the body surface area (BSA) [5]. In SJS, skin detachment is limited to less than 10% of BSA, while in TEN, it is more than 30%. For those skin detachments between 10 and 30% of BSA, they are classified as SJS/TEN overlap. The mortality rate of SJS/TEN is quite high but varies depending on the severity of the disease. It is usually between 1 and 5% in SJS but may be up to 25–30% in TEN [7]. The severity-of-illness score for TEN (SCORTEN) has been widely used to predict mortality of patients with SJS/TEN [8]. The SCORTEN consists of seven variables: (1) age > 40 years, (2) skin detachment > 10% of BSA, (3) heart rate > 120 per minute, (4) presence of malignancy, (5) blood urea nitrogen level > 28 mg/dl, (6) blood glucose level > 252 mg/dl, and (7) blood bicarbonate level < 20 mEq/l. Each item gets one point if it presents. A higher score of the SCORTEN correlates with a higher mortality rate [8].

Histopathological examination is important to confirm the diagnosis of SJS/TEN. It is characterized by numerous apoptotic keratinocytes or forming confluent epidermal necrosis, basal layer vacuolarization, and scarce superficial dermal and perivascular lymphohistiocytic infiltrations [9]. Several mediators have been shown to account for the development of apoptosis of keratinocytes and to be involved in the pathogenesis of SJS/TEN. These include tumor necrosis factor- (TNF-) *α* [10, 11], Fas/Fas ligand [12–14], perforin/granzyme B [15–17], and granulysin [18]. Among them, granulysin exhibits potent toxic effects on keratinocytes and is thought to be the most important mediator in SJS/TEN by far. Granulysin is produced by intraepidermal natural killer (NK) cells and cytotoxic CD8^+^ T-cells in the early phase of SJS/TEN [18]. A rapid test for granulysin has been shown to be an aid for making the diagnosis [19].

In addition to the high mortality rate, several short-term and long-term sequelae have also been reported [20, 21]. Cutaneous and ocular problems were the most common sequelae with an incidence of 44% [22]. The common cutaneous problems include chronic eczema, pigmentary changes, and nail changes. The common ophthalmic complications include dry eye syndrome, chronic conjunctivitis, trichiasis, corneal erosions, and symblepharon [20–22].

### 2.2. Treatment

#### 2.2.1. General Management

Correct identification and prompt withdrawal of the culprit drug are the most important steps in treating patients with SJS/TEN [23]. A useful algorithm has been designed to assess drug causality in SJS/TEN (algorithm of drug causality for epidermal necrolysis (ALDEN)) [24], which could be very helpful to determine the culprit drug.

Supportive care is basically the most important and fundamental treatment for patients with SJS/TEN (Table 1) [25]. Supportive care should include assessment and management of skin wounds, fluid and nutrition status, electrolyte balance, renal and airway function, and adequate pain control [26]. For skin wound care, an antishear handling should be applied to minimize further skin damages. Some experts suggest that the detached skin should be left in situ to act as a biological dressing to protect the underlying dermis, while others argue that the detached skin must be debrided to remove all the potentially infected materials and then covered by biosynthetic dressings [25]. Both approaches are widely used with no good evidence to differentiate which is better. A guideline proposed by the UK experts suggests that debridement may be considered when failure of conservative treatment, presence of wound infection, or delayed healing occurs [27]. Adequate covering of the denuded skin can improve skin barrier function, reduce transepidermal water and protein loss, limit microbial colonization, improve pain control, and promote reepithelialization. Currently, no evidence supports which dressing is superior.

Keeping the fluid balance is an important measurement to prevent end-organ hypoperfusion [27]. It could be monitored daily by a urine output or when necessary by intra-arterial hemodynamic monitoring [27]. A urine output of 0.5–1.0 ml/kg/hr should be maintained [28]. Adequate nutrition support is mandatory because of a hypermetabolic status and large amounts of protein loss in SJS/TEN. It has been suggested to provide up to 20–25 kcal/kg/day in the early phase and up to 25–30 kcal/kg/day in the recovery phase of SJS/TEN by oral intake or nasogastric feeding [27]. Analgesia is necessary and should be adjusted according to the degree of pain. In mild cases, acetaminophen may be adequate, while in severe cases, opiate-based analgesics may be considered [27].

#### 2.2.2. Specific Treatments

There is still a lack of well-designed randomized controlled trial to assess treatment efficacy in SJS/TEN because of rarity of the disease. However, recently, new evidences support that compared to supportive care, some treatments may provide more benefits to the patients. In the following section, we will discuss these commonly used treatments.

*(1) Corticosteroids*. Corticosteroid is by far the most commonly used treatment in SJS/TEN other than supportive care [29]. In the past years, many studies showed noninferiority of systemic corticosteroids compared to the supportive care in treating patients with SJS [30, 31]. Kakourou et al. even found that corticosteroids were significantly associated with decreased fever length and duration of skin lesions [32]. For patients with TEN, there are controversies regarding the usage of corticosteroids. Despite that more studies showed survival benefits on patients with TEN receiving systemic corticosteroids, some studies reported a lack of efficacy or even increased mortality [33, 34]. A high dose of systemic corticosteroids has been shown to be effective in patients with TEN and is recommended by Japanese experts [35]. Araki et al. has reported successfully the use of corticosteroid pulse therapy with a dose of methylprednisolone 500 mg/day for 3 days in 5 patients with TEN [36]. All of them survived. Hirahara et al. have reported similar results in 8 patients with TEN using a dose of methylprednisolone 1000 mg/day for 3 days [37]. A recent published meta-analysis, which collected studies from 1990 to 2012, showed a trend toward survival benefits of systemic corticosteroids compared to supportive care (odds ratio: 0.54; 95% CI: 0.29–1.01) and suggested that systemic corticosteroids are one of the most promising immunomodulating therapies for SJS/TEN [38].

*(2) Intravenous Immunoglobulin*. Intravenous immunoglobulin (IVIG) has attracted much attention since the very first report showing the activation of Fas-Fas ligand in SJS/TEN and the success of treatment with IVIG [12]. Since then, many studies emerged; however, the results were conflicting. Some reports showed survival benefits [39–42], while others did not [43–46]. Dosages of IVIG may have influences on the results of treatment [47]. For those studies with survival benefits, the dosages of IVIG were at least 2.8 g/kg and even up to 4 g/kg. For studies that failed, the dosages of IVIG were mostly 2 g/kg or even lower [47]. Huang et al. performed the first meta-analysis on efficacy of IVIG for the treatment of TEN [48]. They found a significant lower mortality in patients treated with high-dose IVIG compared to those treated with low-dose IVIG (18.9% versus 50%, *P* value = 0.022). However, this trend did not exist after multivariate logistic regression (high versus low dose: odds ratio 0.494; 95% CI: 0.106–2.300, *P* value = 0.369). Lee et al. have reported a retrospective study, which is the largest one till now, analyzing 64 patients with SJS/TEN overlap or TEN treated with IVIG [49]. They found that the use of IVIG does not have survival benefits on SJS/TEN overlap and TEN, even when corrected for IVIG dosages. A recently published meta-analysis also confirmed this observation and showed no differences in mortality when comparing patients receiving IVIG to those receiving supportive care [38].

*(3) Cyclosporine*. Cyclosporine is an immunosuppressive agent inhibiting CD8^+^ cytotoxic T-cells and harboring an antiapoptotic effect through the inhibition of Fas ligand [12] and TNF-*α* [10]. All these cells and mediators play an important role in the pathogenesis of SJS/TEN. It is reasonable to use cyclosporine for the treatment of SJS/TEN. Valeyrie-Allanore et al. conducted a pilot study recruiting 29 patients with SJS/TEN [50]. These patients were treated with cyclosporine 3 mg/kg for 10 days with gradual tapering over 1 month. They found that both mortality rate and progression of skin detachment were lower than expected and suggested a possible usefulness of cyclosporine in SJS/TEN. Recently, Lee et al. reported a retrospective case-control study including 44 patients with SJS/TEN [51]. Among these patients, 24 patients received cyclosporine treatment, while others received supportive care. In the group treated with cyclosporine, 3 deaths were observed. The number of observed death was fewer than that of the SCORTEN-predicted death. Compared to the control group, the standardized mortality ratio of cyclosporine treatment was 0.42 (95% CI: 0.09–1.22). The authors suggested that the use of cyclosporine may improve mortality in SJS/TEN. Recently, Chen et al. performed a meta-analysis on the efficacy of cyclosporine in SJS/TEN [52]. They found that the observed mortality was significantly lower than the SCORTEN-predicted mortality in patients receiving cyclosporine (odds ratio: 0.42; 95% CI: 0.19–0.95) and suggested that cyclosporine harbored a beneficial effect on mortality. Another meta-analysis conducted by Zimmermann et al. also found a similar result showing a significant and beneficial effect of cyclosporine compared with supportive care on mortality [38]. A most recently published study [53] has used three different approaches (case-control, case series, and meta-analysis approaches) to analyze the efficacy of cyclosporine on SJS/TEN. They found that all these three approaches showed consistently a reduction in mortality in SJS/TEN patients receiving cyclosporine. Although the use of cyclosporine in SJS/TEN is not quite popular [4], it seems to be a promising treatment. Further large-scale randomized controlled studies are needed to confirm this observation.

*(4) Anti-TNF-α Agents*. Increased expressions of TNF-*α* in skin specimens [54], in blister fluid, and in serum [17] of SJS/TEN patients justified the strategy of anti-TNF-*α* treatment. With this regard, thalidomide had been chosen as one of the options because of its anti-TNF-*α* property. Wolkenstein et al. had conducted a double-blind, randomized, placebo-controlled trial to evaluate the efficacy of thalidomide [55]. However, it terminated earlier as an unexpected increase in mortality in the patients receiving thalidomide. Nevertheless, the failure of thalidomide did not reject the rationale of anti-TNF-*α* therapy. After the launch of anti-TNF-*α* biologics, several case reports showed positive results of infliximab or etanercept in the treatment of SJS/TEN [56–60]. Paradisi et al. published a case series regarding the use of etanercept in TEN [61]. They recruited 10 consecutive patients with TEN (median SCORTEN: 3, range: 2–6) and treated them with a single subcutaneous injection of 50 mg etanercept. All patients survived and responded well with complete reepithelialization. The median time to healing was 8.5 days. Although it is a preliminary study, the result shows that anti-TNF-*α* therapy may be an effective treatment for SJS/TEN. Further studies are absolutely needed.

*(5) Plasmapheresis*. Plasmapheresis may remove toxic and harmful mediators from the patients and has been shown to provide rapid and dramatic improvement in some reports [62–65]. Narira et al. have demonstrated the usefulness of plasmapheresis in patients who were refractory to conventional therapies and have shown a correlation between disease severity and serum cytokine levels before and after treatment with plasmapheresis [66]. In these patients, serum levels of interleukin- (IL-) 6, IL-8, and TNF-*α* decreased after plasmapheresis. Plasmapheresis is now a recommended treatment option by Japanese experts for patients with TEN who are refractory to high-dose corticosteroids [66].

## 3. Drug Reaction with Eosinophilia and Systemic Symptoms (DRESS)

### 3.1. Basic Characteristics

DRESS, which is also named as drug-induced hypersensitivity syndrome (DiHS) by Japanese experts, is a life-threatening disease presenting with fever, cutaneous eruptions, and internal organ involvement [67]. The mortality rate of DRESS is about 10% [68]. Skin lesions in patients with DRESS have some common features, including an extent greater than 50% of BSA, presences of infiltrative papules and plaques with markedly purpuric change, development of facial edema, and occurrence of desquamation in the stage of resolution [67]. Mucosal lesions may be found in more than 50% of the patients with mouth and lips being the most common site [69]. Systemic symptoms usually present with variable organ/systems involved. Among the hematological abnormalities, eosinophilia is the most common one, being present in 66–95% of the patients, followed by atypical lymphocytosis, which could be found in 27–67% of the patients [69]. In addition, lymphadenopathy can be found in 54% of the patients by physical examinations or image studies [69]. For internal organ involvements, the liver is the most frequently encountered one with a rate of 75–94% of the patients, followed by the kidney, lung, and heart [67]. The duration between the start of the culprits and development of the disease is long with a range usually between 3 and 8 weeks [67]. The list of the causative drugs is long, but most of which are limited to a few categories of drugs, including anticonvulsants, anti-infectious (antibiotics, antituberculosis, and antiviral) agents, sulfonamides, and uric acid-lowering medications [67]. The clinical courses of DRESS usually lasted for more than 15 days with a predilection of protracted and prolonged courses [67]. Waves of recurrence of clinical symptoms may sometimes be encountered possibly accompanied by episodic reactivations of human herpes viruses (HHVs) [70, 71]. Reactivations of HHVs, especially HHV-6, are observed in certain patients during the acute stage and subsequent periods of flare-ups. Therefore, it has been suggested that both antidrug and antiviral immune responses contribute to the development of the disease [67]. In addition to a considerable mortality rate in the acute stage of the disease, there have been certain sequelae reported in the literature [72]. These sequelae included permanent renal dysfunction with a requirement of dialysis, fulminant type 1 diabetes mellitus, thyroid disorders, and autoimmune diseases [72, 73].

### 3.2. Treatment

For treatments of patients with DRESS, there is still insufficient clinical evidence because most of the suggestions are derived from case series or experts' opinions [67]. Immediate withdrawal of the culprit drugs is unsurprisingly the most important thing to do in the management of patients with DRESS. There have been several options of systemic treatments suggested in the literature (Table 1).

#### 3.2.1. Supportive Care Only

Supportive care only may be considered a treatment option for patients with DRESS. A few case series supported this notion. Uhara et al. have reported 12 patients with DiHS who received hydration with or without topical steroids [74]. All the patients recovered well within 7 to 37 days after the withdrawal of the culprit drugs. Ushigome et al. have also presented 17 cases of DiHS treated with only supportive care [75]. All of them recovered smoothly except for those with a higher rate of detectable autoantibodies and a higher rate of autoimmune long-term sequelae. However, the number of patients with DRESS or DiHS treated with only supportive care is still limited. Further studies including a larger number of patients are needed to confirm the observation.

#### 3.2.2. Corticosteroids

Systemic corticosteroids are the mainstay treatment for patients with DRESS. There is still a lack of consensus regarding the dosage and the duration of systemic corticosteroids. A starting dose of 0.5–1.0 mg/kg/day of prednisolone with a gradual tapering over 2-3 months has been suggested by some experts [67]. This approach may reduce the occurrence of disease flare-ups and decrease the probability of the development of autoimmune sequelae [67]. Nevertheless, a prolonged duration of systemic corticosteroid usage may be associated with a higher rate of opportunistic infections and with the possibility of many complications. Funck-Brentano et al. have reported a retrospective study of 38 patients with DRESS [76]. Among these patients, some received supportive care with topical steroids, while others received systemic steroids. The authors found higher rates of infections, septicemia, and the need for intensive care in patients receiving systemic steroid and suggested that systemic steroids should be reserved for those with severe presentations. Thus, individual adjustments are needed for each case based on the severity of the disease and underlying comorbidities. One group of the French Society of Dermatology has recommended that the use of systemic corticosteroids may be considered when 5-fold elevation of serum transaminase levels or involvement of any other organs, such as the kidney, lung, and heart, occurs [77].

#### 3.2.3. Intravenous Immunoglobulin

The results of the use of IVIG in the treatment of patients with DRESS are conflicting. Several studies have reported the successful results [78, 79]. On the other hand, Joly et al. reported their unfavorable experience of using IVIG treatment in 6 DRESS patients [80]. Among them, 5 of the patients had severe adverse effects, with 4 patients requiring systemic corticosteroids due to the adverse effects of IVIG or uncontrolled diseases. Therefore, the authors suggested that IVIG should not be used as monotherapy in treating DRESS syndrome. Obviously, the use of IVIG in the treatment of DRESS needs further investigations.

#### 3.2.4. Other Treatments

Anecdotal reports have shown the treatment effectiveness of several immunosuppressive agents other than those of corticosteroids. These include cyclosporine [81], cyclophosphamide [82], mycophenolate mofetil, and rituximab [67]. Antiviral therapies such as ganciclovir have been proposed in addition to systemic corticosteroids or IVIG to be used in patients with severe disease and confirmation of viral reactivation [77]. However, such treatment should be thoroughly considered by the judgment between benefits and harms.

## 4. Acute Generalized Exanthematous Pustulosis (AGEP)

### 4.1. Basic Characteristics

AGEP is characterized by a sudden onset of at least dozens and often hundreds of sterile, nonfollicular pustules on an edematous erythema with a predilection at the major folds [83]. Sometimes, facial edema, blisters, or atypical target lesions may develop. Mucosal lesions are rare and usually mild. Fever and leukocytosis are commonly accompanied by cutaneous eruptions. Systemic involvements have been reported to develop in less than 20% of the patients with AGEP [84]. Liver involvement is the most common one, followed by the kidney, lung, and bone marrow. Although AGEP may result from viral infections [85], it is primarily a hypersensitivity reaction to drugs. The most strongly associated drugs are pristinamycin, ampicillin/amoxicillin, quinolones, hydroxychloroquine, anti-infective sulfonamides, terbinafine, and diltiazem based on a multinational case-control EuroSCAR study [86]. The latent periods between the drug intake and development of the disease showed two different patterns [86]. For those exposed to antibiotics, the median duration was 1 day, while for those using other medications, the median duration was 11 days. The explanation for these differences is largely unexplored. The prognosis of AGEP is generally very good. Most of the patients recovered without sequelae.

### 4.2. Treatment

The mainstay of treatment for AGEP is the identification and removal of the possible culprit drugs (Table 1) [83]. Recovery and resolution of the skin eruptions usually develop within several days after withdrawal of the culprit drugs [83]. The mean durations between the resolution of the pustules and cessation of the culprit drugs have been reported to be 6 days [87] and 7.6 days [88] in two different studies, respectively. Hospitalization may be required in some patients, especially those with extensive cutaneous eruptions, altered general condition, and systemic involvement. Topical corticosteroid may be used and has been correlated with a decreased median duration of hospitalization [89]. Systemic corticosteroids have been used in some patients with AGEP [88]. However, because of the benign courses in most of the patients with AGEP, the beneficial effects of the usage of systemic corticosteroids need further investigations.

## 5. Generalized Bullous Fixed Drug Eruption (GBFDE)

### 5.1. Basic Characteristics

GBFDE is a rare and severe form of fixed drug eruption (FDE). It is characterized by large areas of well-demarcated erythematous or hyperpigmented patches with blisters or erosions developed soon after administrating the culprit drugs [90]. It exhibits typical features of FDE but may resemble the presentations of SJS/TEN. To differentiate these two diseases is important. One previous study identified that patients with GBFDE had a shorter latent period and less mucosal involvement compared to those with SJS/TEN [90]. The mean duration of the latent period was 3.2 days in GBFDE. Mucosal involvements were only identified in 43% of the patients. Upon histopathological examination, skin specimens of patients with GBFDE showed more eosinophil infiltration and more dermal melanophages. Lesional infiltrates in GBFDE had more dermal CD4^+^ cells including Foxp3^+^ cells, less intraepidermal CD56^+^ cells, and fewer intraepidermal granulysin^+^ cells compared to those in SJS/TEN. The serum level of granulysin in GBFDE was significantly lower than that in SJS/TEN [90]. These features may help to differentiate the two diseases when skin lesions are ambiguous. The common culprit drugs in GBFDE were antibiotics, including cephalosporins, penicillins, and anti-infective sulfonamides, followed by nonsteroid anti-inflammatory drugs [90]. Traditionally, the prognosis of GBFDE is thought be better than that of SJS/TEN. However, a large retrospective case-control study including 58 patients with GBFDE and 170 patients with SJS/TEN matched for age and extent of skin detachment failed to support this traditional concept [91]. The authors found that the mortality rate was slightly but not significantly lower for patients with GBFDE than for controls (22% versus 28%, multivariate odds ratio: 0.6, 95% CI: 0.3–1.4). Although some selection bias may exist in this study, the observation highlights the nature of GBFDE as SCAR might be overlooked before.

### 5.2. Treatment

Currently, there is still a lack of reports regarding the treatment of patients with GBFDE. Just like all other drug reactions, prompt identification and removal of the possible culprit drugs are the most important steps to manage the disease (Table 1). Skin lesions of GBFDE patients usually recover gradually after withdrawal of the causative drugs as that usually seen in patients with conventional FDE. However, for those patients with extensive areas of skin detachment, intensive supportive care should be applied as that used in treating patients with SJS/TEN. Systemic corticosteroids may be used as a treatment option for GBFDE and may be considered effective. Our own unpublished data consisting of 32 patients with GBFDE showed only one death occurring during the acute stage of the disease. Most of these patients were treated with systemic corticosteroids. Nevertheless, due to a lack of sufficient evidence regarding the treatments of GBFDE, further investigations are needed.

## 6. Conclusion

The rarity of SCAR cannot dampen the importance of management of these diseases. All these diseases, including SJS/TEN, DRESS, AGEP, and GBFDE, harbor considerable rates of morbidities and mortalities, which could not be overlooked. However, indeed, the low incidence of SCAR limits the execution of large-scale randomized trials, which in turn, leads to a lack of sufficient clinical evidence in the management of these diseases. Except the existence of some meta-analyses in the treatment of patients with SJS/TEN, for patients with other SCARs, there is a big gap between clinical practice and evidence-based management. Further efforts are needed on these issues to improve the knowledge of SCAR management.

## Figures and Tables

**Table 1:** Treatments for severe cutaneous adverse reactions (SCARs).

SCARs	Comments
*SJS/TEN*	
Supportive care	It is the most important and fundamental treatment and should include assessment and management of skin wounds, fluid and nutrition status, electrolyte balance, renal and airway function, and adequate pain control.
Systemic corticosteroids	They are the most commonly used treatment in SJS/TEN other than supportive care. There are controversies regarding the usage of corticosteroids. There is a trend toward survival benefits of systemic corticosteroids compared to supportive care (odds ratio: 0.54; 95% CI: 0.29–1.01).
IVIG	The results were conflicting. A recently published meta-analysis showed no differences in mortality when comparing patients receiving IVIG to those receiving supportive care.
Cyclosporine	Three recent meta-analysis studies showed a significant and beneficial effect of cyclosporine compared with supportive care on mortality.
Anti-TNF-*α* agents	There is an unexpected increase in mortality in the patients receiving thalidomide. Several case reports and one case series showed positive results of infliximab or etanercept in the treatment of SJS/TEN.
Plasmapheresis	Plasmapheresis may remove toxic and harmful mediators from the patients and has been shown to provide rapid and dramatic improvement in some reports.
*DRESS*	
Supportive care	It might have a higher rate of detectable autoantibodies and a higher rate of autoimmune long-term sequelae. Further studies are needed.
Systemic corticosteroids	They are the mainstay treatment. They may reduce the occurrence of disease flare-ups and decrease the probability of the development of autoimmune sequelae. Individual adjustments are needed.
IVIG	Results are conflicted. It should not be used as monotherapy.
Others	These include cyclosporine, cyclophosphamide, mycophenolate mofetil, and rituximab. Antiviral therapies such as ganciclovir have been proposed in addition to systemic corticosteroids or IVIG in patients with severe disease and viral reactivation.
*AGEP*	
Supportive care	It includes identification and removal of the possible culprit drugs.
Topical corticosteroids	They were correlated with a decreased median duration of hospitalization.
Systemic corticosteroids	The beneficial effects of the usage of systemic corticosteroids need further investigations.
*GBFDE*	
Supportive care	It includes prompt identification and removal of the possible culprit drugs.
Systemic corticosteroids	There is a lack of sufficient evidence.

AGEP: acute generalized exanthematous pustulosis; DRESS: drug reaction with eosinophilia and systemic symptoms; GBFDE: generalized bullous fixed drug eruption; IVIG: intravenous immunoglobulin; SJS: Stevens-Johnson syndrome; TEN: toxic epidermal necrolysis; TNF: tumor necrosis factor.
